# Preservation of methylated CpG dinucleotides in human CpG islands

**DOI:** 10.1186/s13062-016-0113-x

**Published:** 2016-03-22

**Authors:** Alexander Y. Panchin, Vsevolod J. Makeev, Yulia A. Medvedeva

**Affiliations:** Institute for Information Transmission Problems, Russian Academy of Sciences, Moscow, 127994 Russia; Vavilov Institute of General Genetics, Russian Academy of Sciences, Moscow, GSP-1, 119991 Russia; Research Institute for Genetics and Selection of Industrial Microorganisms, Moscow, 117545 Russia; Center for Bioengineering, Research Center of Biotechnology RAS, Russian Academy of Science, Moscow, 117312 Russia; Moscow Institute of Physics and Technology, Moscow Regoin, 141700 Russia

**Keywords:** CpG island, Natural selection, Methylation, Cytosine, Genome-wide substitution rates, CpG

## Abstract

**Background:**

CpG dinucleotides are extensively underrepresented in mammalian genomes. It is widely accepted that genome-wide CpG depletion is predominantly caused by an elevated CpG > TpG mutation rate due to frequent cytosine methylation in the CpG context. Meanwhile the CpG content in genomic regions called CpG islands (CGIs) is noticeably higher. This observation is usually explained by lower CpG > TpG substitution rates within CGIs due to reduced cytosine methylation levels.

**Results:**

By combining genome-wide data on substitutions and methylation levels in several human cell types we have shown that cytosine methylation in human sperm cells was strongly and consistently associated with increased CpG > TpG substitution rates. In contrast, this correlation was not observed for embryonic stem cells or fibroblasts. Surprisingly, the decreased sperm CpG methylation level was insufficient to explain the reduced CpG > TpG substitution rates in CGIs.

**Conclusions:**

While cytosine methylation in human sperm cells is strongly associated with increased CpG > TpG substitution rates, substitution rates are significantly reduced within CGIs even after sperm CpG methylation levels and local GC content are controlled for. Our findings are consistent with strong negative selection preserving methylated CpGs within CGIs including intergenic ones.

**Reviewers:**

Reviewed by: Vladimir Kuznetsov, Shamil Sunyaev, Alexey Kondrashov

**Electronic supplementary material:**

The online version of this article (doi:10.1186/s13062-016-0113-x) contains supplementary material, which is available to authorized users.

## Background

In mammalian genomes cytosines followed by guanines (CpGs) are frequently methylated. The resulting 5-methylcytosines (^5m^C) [1] are prone to deamination and consequent C > T mutations [2, 3]. Frequent cytosine methylation is usually considered as the main cause of a 3-12-fold excess of C > T substitutions in the CpG context [4–7], with *de novo* mutation rates showing an up to 18-fold excess [8]. The main cause of this bias is believed to be a significant underrepresentation of CpG dinucleotides in mammalian genomes, including the human genome [5, 6].

Within genomic regions called CpG islands (CGIs) the frequency of CpG dinucleotides is about 7–10 times higher and the CpG > TpG substitutions rate is about seven times lower than in other regions of the genome [7]. This observation is usually explained by decreased CpG methylation within CGIs. However, although CpG methylation is less common in CGIs, ^5m^CpGs are not as rare within CGIs as thought previously, at least in germline cells, including human spermatozoids [9] and embryonic stem cells [10]. Thus, the question remains whether decreased CpG methylation is in fact sufficient to explain the difference between CpG > TpG substitution rates in CGIs and non-CGI genomic regions or some other mechanisms might be involved. In fact, previously it has been shown that CpG islands are frequently present around the transcription start sites of housekeeping genes [11], suggesting that they might be at least partially preserved by selective pressure for regulatory regions.

Usually, the effect of DNA methylation on CpG > TpG substitution rates (^5m^CpG deamination rate) is evaluated *in silico* from the difference between C > T (G > A) substitution rates in CpG and GpCpH (H = {A,C,T}) contexts [4]:

$$ 5\mathrm{mCpG}\kern0.5em \mathrm{deamination}\kern0.5em \mathrm{rate}\kern0.5em =\frac{\#\left[CpG\kern0.5em >\kern0.5em TpG(CpA)\right]}{\#\left[CpG\right]}-\frac{\#\left[ GpCpH\kern0.5em >\kern0.5em  GpTpH\right]}{\#\left[ GpCpH\right]} $$

This measure exploits the idea that cytosines not followed by guanines are not methylated in mammalian genomes [12]. Yet, it is unclear whether the GpCpH > GpTpH substitution rate provides a good estimate for the unmethylated cytosine substitution rate (C > T). It has been shown that in embryonic stem cells a quarter of all methylated cytosines are found in the CpHpN context [10] making methylation of the first cytosine in the CpHpN context not sporadic. Thus, some cytosines in the GpCpH context are actually methylated and prone to deamination. In addition, local DNA properties such as GC (C + G) content can affect not only the level of cytosine methylation but also the ^5m^C deamination rate itself [4]. Keeping these disadvantages in mind, a more accurate method is desirable to estimate the effect of methylation on CpG > TpG substitution rates.

In our study we question whether the reduced methylation levels of CGIs can fully explain the decreased CpG > TpG substitution rates. To identify CpG > TpG substitutions in the human lineage we reconstructed the ancestral states of human single nucleotide polymorphisms (SNPs) [13]. Since only a small fraction of SNPs has been inherited from the most recent common ancestor of chimps and humans [14] most SNPs can be regarded as substitutions in the human lineage. The direction of these substitutions can be inferred from chimp and orangutan genomic sequences. Using published data on human *de novo* mutations would be a more direct approach [8], however, the observed number of *de novo* mutations in CGIs is currently too small, making the statistical analysis underpowered. This occurs because CGIs cover less than 1 % of the genome [15]. On average one out of 20 dinucleotides (roughly estimated) within CGIs are CpGs and not all of them are methylated.

What methylation data is appropriate for this study? Only germline mutations (mutations in gametes, zygotes, blastomeres, embryoblast cells, epiblast cells, primordial germ cells, and gametogonia) can be inherited, while somatic cell mutations cannot. Therefore, only the methylation patterns in the former cell types are relevant to our study. Germline cells undergo many division cycles on the development path from zygotes to gametogonia, and therefore are likely to accumulate many mutations, including methylation-dependent CpG > TpG mutations. While somatic methylation itself should not affect the observed substitution rates, it can be correlated with mutation rates in regions where somatic and germline methylation profiles are similar [16, 17].

Consistent with previous views [18], a recent study confirmed that the number of mutations in human offspring is highly correlated with their fathers’ age [8], suggesting prevalent accumulation of mutations in the male germline. In addition, sperm cells demonstrate one of the highest methylation levels among the germline cells [17]. This makes sperm cells one of the most promising objects to study methylation-dependant substitution rates [17]. Fortunately, high quality methylation data are available for human sperm [9, 19, 20]. Spermatogonia cells would be an even better choice for the purpose of our study, since they constantly divide by mitosis during the entire male adult life accumulating a perceptible fraction of all germline mutations. Unfortunately, genome-wide methylation profiles of spermatogonia are currently unavailable. Oogonia methylation might also contribute to mutation rates, but such data is also unavailable. Oocytes have been shown to exhibit higher CGI methylation levels than sperm [20], but they do not undergo cell division and thus are not expected to acquire many mutations. In addition, there is available data on methylation in embryonic stem cells (ESC), another type of germline cells undergoing many mitotic divisions [10]. Thus, sperm and ESC data seem to be the most relevant available approximations of methylation patterns in the human germline.

## Results and discussion

### Cytosine methylation in sperm cells affects CpG > TpG substitution rates both within and outside of CpG islands

**Fig. 1:**
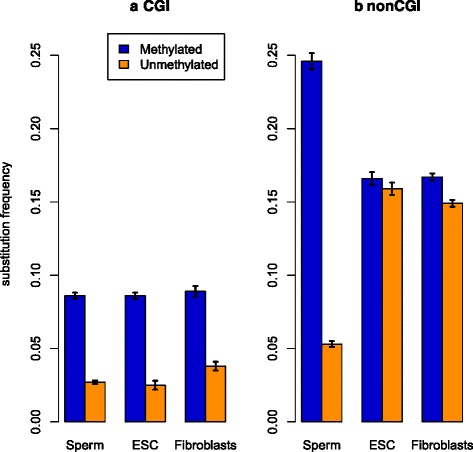
CpG > TpG substitution rates in methylated and unmethylated positions of CGIs (**a**) and non-CGI (**b**) regions. Confidence intervals are calculated according with one-sample *t*-test for deviation from the population mean (α = 0.00001)

CpG methylation in sperm cells was strongly and consistently associated with the increased CpG > TpG substitution rates in both CGIs (Fig. 1a) and non-CGI genomic regions (Fig. 1b). Surprisingly, methylation levels of ESCs and fibroblasts were associated with increased CpG > TpG substitution rates only in CGIs (chi-square test, *P* < 0.001), but not outside of them. This suggests that methylation profiles are more stable within CGIs than non-CGI genomic regions when different cell types are compared. The majority of CpGs in the human genome are found in non-CGI genomic regions. Thus, sperm methylation levels are much better predictors of overall substitution rates in the human genome than methylation levels within the other two cell types.

It could be argued that methylation patterns change in a rather complex manner with several waves of methylation-demethylation events during development, so methylation profiles in other types of germline cells may also affect CpG > TpG substitution rates. On the other hand, the 5-fold increase of CpG > TpG substitution rates in sperm methylated cytosines (Fig. 1) is close to the overall CpG > TpG substitution bias estimates [4, 13, 21, 22], especially if we consider estimates based on similar substitution data [13]). Taken together these observations allow us to conclude that sperm methylation levels are the most appropriate available data to study the genome-wide effects of methylation on TpG > CpG substitution rates. With all this taken into account, sperm methylation data was used in subsequent analysis.

### ^5m^CpG > TpG substitution rates are decreased within CpG islands

**Fig. 2:**
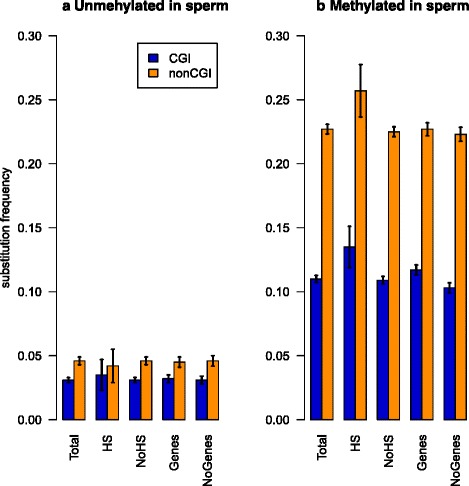
CpG > TpG (**a**) and ^5m^CpG > TpG (**b**) substitution rates in sperm in various genomic locations. Confidence intervals are calculated according with one-sample *t*-test for deviation from the population mean (α = 0.00001)

If the reduced level of CpG methylation in CGIs is the main cause of reduced CpG > TpG substitution rates in CGIs we would expect that CpGs with the same methylation levels in CGIs and non-CGI genomic regions would have similar CpG > TpG substitution rates. On the contrary, even after controlling sperm methylation levels and local GC content, we still observed an approximately 2-fold reduction (CGI impact = 2.1, *P* < 0.001, see Methods: *Comparison of CpG > TpG substitution rates and CGI impact*) of ^5m^CpG > TpG substitution rates in CGIs (Fig. 2b). The protective effect of CGIs appeared to be even stronger for ^5m^CpGs than for unmethylated CpGs (Fig. 2a).

It is worth noting that the criteria for CGI identification are rather arbitrary, e.g. some minimal CGI length is usually required. Short CpG islets could perform the same function as long CpG islands [23] yet they would not be classified as CGIs by annotating software. Under the assumption, that the reduction of ^5m^CpG > TpG substitution rates is related to some yet unclear function of CGIs, it is obvious that there should be ^5m^CpGs dinucleotides located outside of annotated CGIs that perform similar functions and have their ^5m^CpG > TpG substitution rates reduced. Thus, the measured CGI impact is likely to be underestimated.

Since we controlled for local GC content and sperm methylation levels, we hypothesized that some other factors related to CGIs contributed to the observed reduction of ^5m^CpG > TpG substitution rates in CGIs. This factor could either reduce mutation rates in ^5m^CpG or increase negative selection preserving such positions. Below, we addressed both possibilities and tested several possible explanations of this effect.

### Biased gene conversion does not explain the decreased ^5m^CpG > TpG substitution rates within CGIs

The reduction of ^5m^CpG > TpG substitution rates within CGIs could be a result of a biased gene conversion (BGC) that according to some studies plays an important role in the origin of CGIs [24]. In mammals gene conversion occurs between double-stranded DNA and is biased towards increasing GC content [25], most likely placing C or G when a mismatched pair is observed. If gene conversion is more efficient within CGIs, CpG > TpG mutations would be reverted more frequently within CGIs and the number of observed substitutions in CGIs would be reduced.

Since BGC takes place during DNA recombination, in theory it should be more effective in recombination hotspots, resulting in relatively lower local ^5m^CpG > TpG substitution rates in such regions. However, we observed ^5m^CpG > TpG substitution rates to be slightly higher within CGIs in recombination hotspots (Fig. 2a). We observed that ^5m^CpG > TpG substitution rates in non-CGI genomic regions were consistently higher than ^5m^CpG > TpG substitution rates within CGIs both within and outside of the recombination hotspots. If the decreased ^5m^CpG > TpG substitution rates in CGIs were explained by the increased efficiency of BGC within CGIs, one would expect a more pronounced reduction within regions with higher recombination rates. On the contrary, CGI-associated reduction of ^5m^CpG > TpG substitution rates was slightly more pronounced in regions far from recombination hotspots (CGI impact = 2.1 vs 1.9, Fig. 2a, *P* < 0.001). This discards BGC as an explanation for the comparative reduction of ^5m^CpG > TpG substitutions in CGIs.

Our analysis of BGC was based on recombination data and should be taken with caution. In many cases, the ability to recognize recombination events depends on the existence of variations between homologous genomic regions undergoing recombination. If two homologous DNA sequences are identical and a recombination event occurs between them, this recombination event is hard to detect, leading to an underestimation of the recombination hotspots number.

Recently it has been shown that CpG sites located in the late replicating regions tend to accumulate more mutations [26, 27]. However, Chen et al. [27] have reported that the increase in the CpG > TpG substitution rate is probably a by-product of increasing frequency of cytosine methylation from early- to late-replicating regions in the germline. Thus, we doubt that variations in replicating timing as well as BGC can explain the observed reduction of ^5m^CpG > TpG substitution rates in CGIs after methylation levels in germline are controlled for.

### ^5m^CpG > TpG substitution rates are decreased even within intergenic CGIs

Another factor, which could decrease substitution rates within CGIs, would be negative selection protecting CpGs from elimination. If the fraction of CpG dinucleotides under negative selection were higher within CGIs, the observed substitution rates would be lower in CGIs [28]. CGIs are frequently located near protein-coding genes therefore are more likely to experience negative selection due to their overlap with protein-coding regions and sequences that regulate gene activity. To control for gene-associated negative selection, we performed a separate analysis of ^5m^CpG > TpG substitution rates in genomic regions located far from genes (>1000 bp away from known genes). Still, we observed a reduction of ^5m^CpG > TpG substitution rates within CGIs located far from genes (Fig. 2a), which supported the idea that gene-associated negative selection does not explain the decreased ^5m^CpG > TpG substitution rate in CGIs. Moreover, the difference between ^5m^CpG > TpG rates in CGI and non-CGI genomic regions was more pronounced for CpGs located far from genes, suggesting a limited role of gene-related selection in protection of CpGs in CGIs. If negative selection did contribute to the reduction of ^5m^CpG > TpG substitution rates in CGIs, it was selection associated with CGIs themselves, rather than with gene flanking regions.

It is noteworthy that the majority of CGIs located far from protein-coding genes overlap with promoters for various ncRNAs [29, 30]. Therefore, they might undergo negative selection, associated with the preservation of these regions. In addition, the human genome can contain non-annotated lncRNA genes, which were recently demonstrated to be under selection to retain high GC content and splicing enhancers within their exons [31]. On the other hand, our control set made of short GC- and CpG-rich regions, may also include functional and therefore conserved segments [23]. Previously we have shown that long non-coding RNA promoters although usually lacking CGIs still demonstrated strong GC bias suggesting functional role of short GC rich tracks located near lncRNA promoters even not overlapping with CGIs [32]. We addressed the role of regulatory sequences in reducing ^5m^CpG > TpG substitution rates in detail.

### Role of transcriptional factor binding sites and epigenetic mechanisms in ^5m^CpG > TpG substitution rates

Negative selection can clean out mutations that affect the affinity of DNA binding proteins in regulatory DNA regions. This provides a compelling explanation of the reduced substitution rates in CGIs. Several reports have shown that CGI DNA fragments integrated into either gene deserts or genomic regions lacking transcription start sites can maintain their unmethylated status and recruit sequence-specific transcription factors (TF), such as SP1, CTCF or CXXC1/Cfp1, or Polycomb-group proteins [25, 33, 34]. Similarly, negative selection can specifically affect methylated cytosines. Methyl-binding proteins (MBD), such as MBD2 and MeCP1 targeting ^5m^CpG, are known to play an important role in gene repression [35], thus substitutions within MBD binding sites might also be under negative selection. This idea is supported by the observation that MBD2 binding sites in vitro are co-localized with transcriptional start sites [36], which often overlap with CGIs. Additionally, binding sites occupied by a protein molecule might be less prone to mutations due to some stereochemical reasons or to chromatin changes caused by MBD-proteins.

**Fig. 3:**
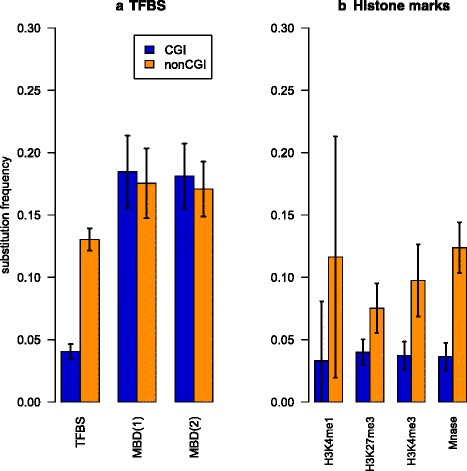
^5m^CpG > TpG substitution rates in conserved TFBS, regions of MBD binding (**a**) regions of nucleosome occupancy and histone modifications (**b**) Confidence intervals are calculated according with one-sample *t*-test for deviation from the population mean (α = 0.00001). All regions are located at least 1000 bp from gene boundaries

To test whether transcriptional factor binding sites (TFBS) affect ^5m^CpG > TpG substitution rates, we specifically studied the role of conserved TFBS and particularly MBD2 proteins binding sites. To exclude effects of selection on protein-coding genes, we focused on areas located far from genes. Figure 3a shows that conserved TFBS demonstrated reduced ^5m^CpG > TpG substitution rates in both CGIs and non-CGI regions as compared to regions without conserved TFBS. Surprisingly, MBD2 binding sites did not protect ^5m^CpG from substitutions. Partially this could be due to the data type (Chip-seq peaks), which did not allow us to accurately locate particular TFBS. Yet, for TFBS regions ^5m^CpG > TpG substitution rates were reduced in CGIs even at a greater degree than generally in the genome (CGI impact = 3.6, *P* < 0.001). Thus the high frequency of conserved TFBS or binding sites for MBD2 proteins in CGIs was unlikely to explain the reduced ^5m^CpG > TpG substitution rates. In a sense, this agrees with the observation that TFBS in general avoid CpG with functional methylation [37, 38].

Other epigenetic mechanisms may also contribute to changes in CpG > TpG substitution rates. Hydroxymethylcytosine (^5hm^C), a result of oxidation of ^5m^C frequently present at least in ESC [39], cannot be distinguished from ^5m^C neither by bisulfate conversion nor by enzymatic techniques [40, 41]. To the best of our knowledge the effect of hydroxymethylation on CpG > TpG substitution rates is not explicitly estimated yet, however it has been shown that ^5hm^C may be a first step to DNA demethylation [42]. One may assume that oxidation of ^5m^C increases the probability of cytosine restoration and, therefore, reduces observed ^5m^CpG > TpG substitution rates. Also recently it has been shown that hydroxymethylation increases C > G transversion rates [43]. This effect can also contribute to the observed differences in the substitution rates. The potential contribution of hydroxymethylation to ^5m^CpG > TpG substitution rates is supported by recent observation of a high ^5hm^C frequency within CGIs [44]. Unfortunately, the unavailability of data on ^5hm^C in human sperm prevented us from testing this hypothesis directly.

Several enzymes (at least ACIDA, APEX, APOBEC group proteins, PRDM14, TET group enzymes, TEFT, TDG) appear to be involved in DNA demethylation (listed from [45]). At least PRDM14 has sequence specific preferences of DNA binding (according to http://hocomoco.autosome.ru/; [46]), and the motif can be present in CGIs and outside of them with different frequencies, yet it is probably the most indirectly related protein to DNA demethyaltion and we doubt that it can contribute much to the methylation-dependant mutation frequencies. However if it is shown one day that some of DNA demethylating enzymes demonstrate sequence specific binding properties it should be investigated in detail whether differential CGI/nonCGI binding of such proteins can be responsible for decreased C > T substitution rates in CGIs.

Chromatin state may also affect the substitution rates, changing the availability of DNA to mutagens or the repair system. ^5m^CpG sites in CGI may undergo stronger negative selection because of increased mutability [47] in regions of open chromatin. In addition, CGI promoters demonstrate a distinct chromatin structure as compared to non-CGI promoters [48]. We studied ^5m^CpG > TpG substitution rates and CGI impact in regions occupied by nucleosomes and regions containing histone modifications in sperm. Figure 3b shows reduced levels of ^5m^CpG > TpG substitutions within both areas of activation (H3K4me1 and H3K4me3) and repression (H3K27me3) histone marks as well as within regions occupied by nucleosomes, as compared to the regions for which such modifications were not exhibited. One can hypothesize that regions of chromatin modifications are associated with some additional selection on CpGs. The greatest reduction in ^5m^CpG > TpG substitution rates was observed within regions with H3K27me3, which was in line with previous observation that hyperconserved CpG domains are linked to Polycomb-binding sites [49]. Despite evidence of additional selection due to functional regulatory elements, the CGI impact on ^5m^CpG > TpG substitution rates remained and could not be explained by higher frequency of studied functional regulatory elements in CGIs.

### The frequencies on derived alleles for C/T and G/A polymorphisms are decreased for methylated CpGs in CGIs as compared to methylated non-CGI controls

Using allele frequency data obtained within the 1000 genome project (see Methods: Allele frequency analysis) we evaluated the hypothesis that there is excessive negative selection on methylated CpGs within CGIs as compared to non-CGI genomic regions. The prediction of this hypothesis is that smaller allele frequency values should be observed for derived T and A alleles within CGIs. We observed a highly significant although small effect in support of this hypothesis. Out of two sets of 241526 methylated CpG dinucleotides 16844 and 41360 contained polymorphisms for the CGI and non-CGI datasets respectively. The average derived allele frequency were 0,025 and 0,029 respectively and the median values were 0.0018 and 0.0023 respectively (*P* = 2.2e-16, Wilcoxon rank sum test). Similar median values (0.0018 and 0.0023 respectively) were obtained for derived allele frequencies for CGI and non-CGI intergenic regions, thus the effect was not due to selection on the gene level.

### Other considerations

It is widely accepted that nucleic acids can form non-canonical structures (hairpins, triplexes, R-loops, G- quadruplexes and others). Such structures in DNA can mediate substitution rates affecting subsequent DNA replication and repair efficiency (reviewed in [50]). An R-loop, a three-stranded nucleic acid structure, composed of a DNA:RNA hybrid, leaves the non-transcribed DNA strand unpaired, which in turn can lead to increased substitution rates. A recent work suggested a link between R-loop formation and activation-induced deaminase (AID) activity [51]. AID proteins in humans may create mutations in DNA by deamination of the cytosine (and therefore a U:G mismatch repair into T:A) or can contribute to DNA demethylation if U:G mismatch is repaired into unmethylated C:G. In this regard, a detailed study of specific DNA structures in CGIs would be highly appreciated.

## Conclusions

Cytosine methylation in human sperm cells is strongly associated with increased CpG > TpG substitution rates. Differences in sperm methylation levels, local GC content, negative selection associated with DNA protein coding or regulatory function as well as biased gene conversion to the best of our knowledge do not explain the protective effect of CGI (CGI impact) on ^5m^CpG > TpG substitution rates. Although it can be hypothesized that the ^5m^CpG > TpG substitution rate itself is lower within CpG islands due to reduced mutation rates, our data suggests strong negative selection acting within CGIs. In any case, we can conclude that the likelihood of nucleotide substitutions, including those associated with diseases, depends on the presence of CGIs whether methylated or not.

## Methods

### Methylated and unmethylated cytosines

Sperm methylation data was obtained from [19]. Both available replicates where combined. ESC and fibroblast methylation data was obtained from [10]. Positions covered by less than 10 reads were excluded from the study. We considered a cytosine as unmethylated if none of the reads contained a methylated cytosine. For cytosines methylated in at least one read the level of methylation was estimated as the fraction of methylated reads among all reads.

### CpG > TpG substitutions in the human lineage

To obtain a subsample of CpG > TpG and CpG > CpA substitutions we reconstructed ancestral states of human bi-allele SNP variants that have been aligned to the chimpanzee (*Pan troglodytes*) and orangutan (*Pongo pygmaeus*) genomes (SNP 138, UCSC genome browser mapping, hg19, track: snp138OrthoPt4Pa2Rm3, [20]). We selected only those human bi-allele CpG/TpG and CpG/CpA variants that were aligned with both chimpanzee and orangutan genomes, and where the cytosine/guanine variant was present in both outgroups. We used two outgroups because a single outgroup might be insufficient for a reliable reconstruction of ancestral states [52] due to misinferences caused by high CpG > TpG substitution rates. We assumed that such bi-allele variants resulted from CpG > TpG/CpA substitutions in the human lineage [14].

### CpG islands

Most contemporary methods for CGI identification use DNA sequences masked from repeats prior to CGI prediction, so the resulting set of CGIs usually belongs to the non-repetitive part of the analyzed genome. This is also true for CGIs available in the UCSC genome browser [http://genome.ucsc.edu/cgi-bin/hgTrackUi?hgsid=483112495_cDKZds3Z5LzftC3guOlNqFlNbqor&c=chr19&g=cpgIslandExt], identified with an algorithm by G. Miklem and L. Hillier based on the criteria established in [53]. However, repetitive elements may also function as promoters for noncoding RNAs [29]; therefore, repeat-associated CGIs can have functions similar to those of CGIs associated with coding genes promoters. For this reason, we applied the search algorithm to the complete human genome without masking any sequences. However, only those CpG dinucleotides were included in our samples and controls that were aligned with the chimp and orangutan genomes and for which ancestral state reconstruction was possible and for which methylation data was available.

### Case control analysis

For each of the three methylation datasets (sperm, embryonic stem cells and fibroblasts) we performed two case control comparisons. In the first analysis we compared C > T/G > A substitution rates between methylated and unmethylated CpG dinucleotides while controlling for local GC content, since GC content is largely responsible for DNA methylation levels [54]. In the second analysis we compared C > T/G > A substitution rates between CpG dinucleotides in CGIs and non-CGI genomic regions while controlling both methylation levels in sperm and local GC content (Additional file 1: Figure S1). The first comparison was done separately for CGIs and non-CGI genomic regions. The second comparison was done independently for methylated and unmethylated CpGs.

In all comparisons we created sample and control sets of equal size. Each CpG from the sample set had a matching CpG in the control set with the same GC content in a 250 bp window, located at the same chromosome (thus accounting for chromosome dependent substitution bias; for example, the human Y chromosome is more prone and the X chromosome is less prone to point substitutions than the rest of the genome [55]). When comparing methylated CpGs we additionally required similar degrees of methylation between CpGs (±5 %). The degree of methylation was calculated as the fraction of reads indicating methylated cytosines from all aligned reads. The order of CpGs in lists from which sample and control CpGs were selected was randomized prior to the analysis, to ensure random selection of control CpGs.

### Comparison of CpG > TpG substitution rates and CGI impact

Our data did not allow us to estimate the genuine mutation rate (i.e. the number of CpG > TpG mutations per generation or another time unit). However, we were able to compare substitution rates between the control and sample sets of CpG dinucleotides. The number of polymorphic sites results from the equilibrium of *de novo* mutation accumulation and their elimination. Assuming low or evenly distributed selective pressure the observed number of substitutions should be proportional to the *de novo* mutation rate [28]. Thus one can measure the CpG > TpG substitution rate as the fraction of CpG dinucleotides that have undergone CpG > TpG substitutions in the human lineage. We estimated the impact of the presence of CGI on the substitution rates in a way similar in logic to that used in [47]:

CGI impact = (Number of CpG > TpG/number of CpG) outside CGI/(Number of CpG > TpG/number of CpG) within CGI.

The actual numbers of CpGs used for the analysis are present in Additional file 2: Table S1. We used chi-square tests to calculate the statistical significance of the CGI impact.

### Recombination rate

We took coordinates of recombination hotspots from [56]. We considered regions to have low recombination rates if they were located further than 1000 bp from any hotspot.

### Negative selection in gene-associated regions

We used the knownGene table from the UCSC genome browser [http://genome.ucsc.edu/cgi-bin/hgTrackUi?hgsid=209406589&c=chr21&g=knownGene] to prepare subsamples of CpGs located near and away from genes. We considered a CpG to be located far from genes if it was more than 1000 bp away from the boundaries of any gene (its transcription start site and its termination site).

### Allele frequency analysis

We acquired a set of C/T and G/A bi-allele variants from hg19 1000 genomes dataset available at (http://hgdownload.cse.ucsc.edu/gbdb/hg19/1000Genomes/[57]). SNP allele frequency was extracted for those SNPs that were located within the methylated CpG dinucleotides of our datasets. We compared the median of the novel SNP variant allele frequencies between the control and sample datasets using Wilcoxon rank sum test with continuity correction.

### Transcription factor binding sites and chromatin data

We used data on H3K27me3, H3K4me3 and Histone Mnase from the work of Hammoud, Nix et al. [58], data on H3K4me1 from Hammoud, Low et al. [59] and data on MBD binding sites from the work of Illingworth et al. The coordinates were converted to hg19 using liftOver [https://genome.ucsc.edu/cgi-bin/hgLiftOver]. We considered only regions located more than 1000 bp away from the boundaries of any gene.

All human data used in this work were taken from publically available sources. The papers describing these data with all appropriate ethical statements are already published elsewhere [10, 19, 20, 30, 56, 58, 59].

## Response to the reviewers

### Reviewer 2 (Vladimir Kuznetsov)

DNA methylation occurs at CpG dinucleotides; it is a dynamic epigenetic regulation mechanism in mammalian genomes. The methylated CpG dinucleotides can lead to a high rate of C to T substitution at these sites. It is known that a high mutation rate can be observed in methylated CpG sites. But in CpG islands (CGIs), C to T substitutions rate is much lower compare to other CpG regions of the genome. In this study authors question whether the reduced methylation levels of CGIs can explain the decreased C to T substitution rates. They selected the experimental data for sperm because previous findings suggested prevalent accumulation of mutations in male germline cells and also demonstrated the highest methylation levels among the cells. They showed that cytosine methylation in sperm is strongly associated with increased C to T substitution rates in the whole genome, but in embryonic stem cells the association was weak. Unexpectedly, in sperm cells, C to T substitution rates was low in CGIs but count not be explained by decreased methylation. They proposed that there was strong negative selection acting within CGIs. This study provides new evidence of the likelihood of C to T substitutions depends on the presence of CGIs whether methylated or not. It should be useful for the scientists actively working in the field of epigenetics and mutation and other related disciplines. However, more detail information, more analyses should be performed.

Main Comments and Suggestions

• In the first part of the results (page 4, part 1), the authors claimed that sperm methylation levels were much better predictors of overall substitution rates in the human genome than methylation levels within the other two cell types. Based on this analysis, are there any different/similarity between group of genes containing CGI/nonCGI in sperm and other two cell lines? More analysis is required such as a. Gene Ontology analysis of gene containing CGI and nonCGI. b. DNA motif finding of the regions containing CGI with C to T substitution in sperm compare to other two cell lines. c. The connection of genome architecture and CGI with C to T substitution e.g. C to T substitution rate in bidirectional promoter compare to unidirectional promoter.

Response: *We truly appreciate reviewer’s interest and careful reading of our work. Yet some of the suggestions remain unclear for us. First of all, groups of genes containing CGI/nonCGI are the same in all cell lines, since in our work the CGI annotation is based on DNA sequence only. There are other approaches to define CGIs, mostly using unmethylated co-localized clusters of CpGs in a particular cell type of interest, although if we defined CGIs in this manner the analysis performed in our paper would not be possible, since by definition there are no methylated CpG dinucleotides in such CGI.*

Functional analysis of genes containing CGIs is a very important question and it has been addressed before. To avoid any confusion we added the appropriate statement to the introduction.

“Thus, the question remains whether decreased CpG methylation is in fact sufficient to explain the difference between CpG > TpG substitution rates in CGIs and non-CGI genomic regions or some other mechanisms might be involved. In fact, it has been previously shown that CpG islands are frequently present around the transcription start sites of housekeeping genes [PMID:26512062], suggesting that they might be at least partially preserved by selective pressure for regulatory regions”.

The question of how local motifs may affect mutational rates was addressed in detail in our work [PMID:21718472]. It has been shown that while CpG context contributes a lot to C > T mutation bias, the effects of local up-to-four nucleotide context around CpGs is relatively small. Yet, this conclusion was obtained by analyzing genome-wide substitution rates and not in a particular cell line. It is a possibility that methylation occurs in different local contexts in different cell types. Also mutation may occur with different frequencies in such methylated contexts in various cell lines. Yet, since we expect these effects to be relatively small as compared to the effect of CpG, we believe that rough estimation will not help. At the same time an accurate analysis of context effects would require (1) careful correction for the effects of small motifs (in particular CpG) inside the longer ones; (2) higher coverage of the sequencing to cover the majority of CpGs n the genome; and therefore lays beyond the scope of the current study.

Genome architecture can influence C > T mutation rates as well as it can be under selection preserving some DNA sequence patterns. There are several layers of genome architecture that can be involved – two-dimentional (such as genes organization, for example bi-directional promoters) and three-dimensional (such as chromatin organization). We addressed the effects of three-dimentional architecture to some extend and in the updated version of the paper we explicitly explained the motivation to do so.

“In addition, CGI promoters demonstrate a distinct chromatin structure as compared to non-CGI promoters [48]. We studied ^5m^CpG > TpG substitution rates and CGI impact in regions occupied by nucleosomes and regions containing histone modifications in sperm”.

Indeed, the two-dimensional organization can also contribute to substitution rates. The problem with bi-directional promoters is that they are hard to define, since nowadays it is widely believed that the majority of the promoters show bi-directionality [PMID:19377478] at least to some extend and consist of mRNA-ncRNA pair [reviewed, for example, in PMID:26578749] as compared to the previous studies where only protein-coding genes were taken into account. On the other hand, we demonstrated that the major effect of the decreased substitution rates of C > T in CGI remained intact even in CGI located far away from genes, suggesting the same effect outside the promoters. Considering this, we believe that the two-dimensional gene organization is beyond the scope of this study.

• In page 5, topic: 5mCpG > TpG substitution rates are decreased even within intergenic CGIs

• The substitution rate and methylation level should be shown in detailed genomic region such as promoter, enhancer, exonic, intronic, gene terminal and intergenic regions. A previous study by authors demonstrated that many CGIs located far from transcription start sites of any protein coding gene have transcription initiation activity and display Sp1 binding properties (Medvedeva et al., 2010, BMC Genomics). In exons, overlapping with these CGIs, the substitution rate of CpG containing codons is decreased (Medvedeva et al., 2010, BMC Genomics). Therefore, demonstration of the associations in detail should provide more reliable and accurate results.

Response: *In this work, we wanted to eliminate the selection on CGI coming from protein-coding genes sequences and their well-known regulatory region as far as possible. We understand that it is not an easy task, since a lot of yet-undetermined genomic regions, for example, enhancers, can play a regulatory role therefore being under selection. The problem with enhancers is that they are very cell types specific and are not fully annotated so far. Even the best methods to computationally predict enhancers (based on genome-wide experimental data, for example PMID:25678556) can be validated only in about half of the cases. Yet we believe that enhancers, being usually AT-rich, should not contribute to CGI-based selection much. We tried to address the issue of eliminating gene-based selection in more detail.*

“It is noteworthy that the majority of CGIs located far from protein-coding genes overlap with promoters for various ncRNAs [PMID:20085634; PMID:20885785]. Therefore, they might undergo negative selection, associated with the preservation of these regions. In addition, the human genome can contain non-annotated lncRNA genes, which we recently demonstrated to be under selection to retain high GC content and splicing enhancers within their exons [PMID:25589248]. On the other hand, our control set made of short GC- and CpG-rich regions, may also include functional and therefore conserved segments [PMID:20500903].”

We also performed an additional test to find if there is a selection pressure on methylated CpG positions on CGI islands located far from known genes (see ***The frequencies on derived alleles for C/T and G/A polymorphisms are decreased for methylated CpGs in CGIs as compared to methylated non-CGI controls***)

Some important points should be added in the discussion • G-rich in many CGIs are prone to form noncanonical DNA structures such as R-loop forming structures. R-loops could be considered as a factor that may contribute in demethylation process. Previous study by Wongsurawat et al., 2012, NAR, suggested that R-loop forming DNA sequences could be the target of demethylation by AID (activationinduced deaminase).

Response: *We fully agree with the reviewer and added the following text regarding the issue*

“It is widely accepted that nucleic acids can form non-canonical structures (hairpins, triplexes, R-loops, G-quadruplexes and others). Such structures in DNA can mediate substitution rates affecting subsequent DNA replication and repair efficiency (reviewed in [50]). An R-loop, a three-stranded nucleic acid structure, composed of a DNA:RNA hybrid, leaves the non-transcribed DNA strand unpaired, which in turn can lead to increased substitution rates. A recent work suggested a link between R-loop formation and activation-induced deaminase (AID) activity ([51]). AID proteins in humans may create mutations in DNA by deamination of the cytosine (and therefore a U:G mismatch repair into T:A) or can contribute to DNA demethylation if U:G mismatch is repaired into unmethylated C:G. In this regard, a detailed study of specific DNA structures in CGIs would be highly appreciated.”

• DNA repair enzyme thymine DNA glycosylase (TDG) which can reduce the rate of C to T substitution should be also discussed.

Response: *We added the following text to the discussion.*

“Several enzymes (at least ACIDA, APEX, APOBEC group proteins, PRDM14, TET group enzymes, TEFT, TDG) are reported to be involved in DNA demethylation (listed from [45]). At least PRDM14 has sequence specific preferences of DNA binding (according to http://hocomoco.autosome.ru/; [46]), and the motif can be present in CGIs and outside of them with different frequencies, yet it is probably the most indirectly related protein to DNA demethyaltion and we doubt that it can contribute much to the methylation-dependant mutation frequencies. However if it is shown one day that some of DNA demethylating enzymes demonstrate sequence specific binding properties it should be investigated in detail whether differential CGI/nonCGI binding of such proteins can be responsible for decreased C > T substitution rates in CGIs”.

• To discuss the limitations of your approach.

• What kind of the experimental studies should be carrying out to validate your major results?

One of the main limitations of our approach is that we do not take into account the methylation profiles of a number of other germ cell types, most importantly spermatogonia cells, methylation data for which was not available. Different methylation patters in spermatogonia and other cells (such as oogonia, oocytes e.t.c) could also influence the mutation rates in CGIs and non-CGI genomic regions. Our data, however, suggests that methylation data of sperm cells is a good predictor of substitutions in the human lineage. Oocyte data is unlikely useful, since these cells undergo a small number of divisions, and thus their methylation patters are unlikely to contribute much to the patterns on mutations.

Another limitation is that we used substitution data instead of de novo mutation data for our analysis. Unfortunately, there is still not enough de novo mutation data to perform a robust analysis. In the future it would be interesting to analyze the correspondence between de novo mutation rates and methylation patterns in CGIs and non-CGI genomic regions directly.

We made appropriate changes in the text to clarify our thoughts on the matter:

“Using published data on human *de novo* mutations would be a more direct approach [8], however, the observed number of *de novo* mutations in CGIs is currently too small, making the statistical analysis under-powered. This occurs because CGIs cover less than 1 % of the genome [15]. On average one out of 20 dinucleotides (roughly estimated) within CGIs are CpGs and not not all of them are methylated.”

“What methylation data is appropriate for this study? Only germline mutations (mutations in gametes, zygotes, blastomeres, embryoblast cells, epiblast cells, primordial germ cells, and gametogonia) can be inherited, while somatic cell mutations cannot. Therefore, only the methylation patterns in former cell types are relevant to our study. Germline cells undergo many division cycles on the development path from zygotes to gametogonia, and therefore are likely to accumulate many mutations, including methylation-dependent CpG > TpG mutations. While somatic methylation itself should not affect observed substitution rates, although it can be correlated with mutation rates in regions where somatic and germline methylation profiles are similar [16, 17].

Consistent with previous views [18], a recent study confirmed that the number of mutations in human offspring is highly correlated with their fathers’ age [8], suggesting prevalent accumulation of mutations in the male germline. Also sperm cells demonstrate one of the highest methylation levels among the germline cells [17]. This makes sperm cells one of the most promising objects to study methylation-dependant substitution rates [17]. Fortunately, high quality methylation data are available for human sperm [9, 19, 20]. Spermatogonia cells would be an even better choice for the purpose of our study, since they constantly divide by mitosis during the entire male adult life accumulating a perceptible fraction of all germline mutations. Unfortunately, genome-wide methylation profiles of spermatogonia are currently unavailable. Oogonia methylation might also contribute to mutation rates, but such data is also unavailable. Oocytes have been shown to exhibit higher CGI methylation levels than sperm [20], but they do not undergo cell division and thus are not expected to acquire many mutations. Also, there is available data on methylation in embryonic stem cells (ESC), another type of germline cells undergoing many mitotic divisions [10]. Thus sperm and ESC data seem to be the most relevant available approximations of methylation patterns in the human germline.”

Technical part

• Authors may simplify the case control analysis step into flowchart.

Response: *Done. Now the flowchart in provided on the Additional file**1**: Figure S1.*

In Fig. 1, it’s not clear how error br came from. It should be mentioned in the flowchart.

Response: *We a added the explanation to the Figures legends, since the error bars represent a confidence interval based on theoretical distribution and was not obtained during the case–control data analysis.*

• Authors should explain the difference of technique that obtains methylation data of Sperm methylation data [17] and ESC and fibroblast methylation data [9]. Since these two data sets were conducted differently.

The bar plot below show total number of CpG > TpG substitution were retrieved from ESC, Fibroblasts, and Sperm in Additional file 2: Table S1. We reanalysed data and found that number of CpG > TpG substitution within CGI of ESC and Fibroblasts are relatively smaller than those outside CGI. In contrast, the number of CpG > TpG substitution within CGI of Sperm is 3 times higher. We are not sure that the result in Fig. 1b is consequent on technical bias (such as bias selection of CpG > TpG substitution outside CGI in Sperm data).

Response: *In our previous manuscript version, Additional file**2**: Table S1 incorrectly contained estimates of “CGI impact” for the “Case/control analysis of the methylation impact” sub-table. In this sub-table we compared the CpG > TpG substation rate differences between methylated and unmethylated CpGs for three different cell types. Control unmethylated CpGs with matching local GC content were selected for the methylated CpGs and this was done in a similar way for all three types of cell lines we analyzed. Substitution rates in CGIs and non-CGI genomic regions should not be compared in this sub-table, because the sets are not case/control matched. We have now replaced the misleading “CGI impact” values with “Not applicable” text for the sub-table.*

Substitution rates between CGIs and non-CGI genomic regions were compared only for sperm cells (see Sub-table Case/control analysis of the CGI impact). Here CpGs from CGIs and non-CGI genomic regions are case/control matched. In all cases, substitution rates are higher in non-CGI genomic regions, comparing to CGIs.

We would also like to note that we found another error in the previous version of this table: the total number of CpGs was presented incorrectly. We have fixed this issue. This did not affect the CGI impact values or substitution rate values, which were correct from the beginning.

One more thing that we would like to emphasize, is that the experimental methylation data for sperm cells and the two other cell types were obtained by different research groups with certain differences in their workflow methodology. One of the limitations of our claim that the sperm cell methylation rates are better predictors of substitution rates is that we cannot properly take into account the minor differences in the methods used in the different experiments. However, we believe that since the methods are similar in principle (Bisulfite sequencing), bias is unlikely.

### Reviewer 3 (Shamil Sunyaev)

Overall, thus manuscript is of substantial interest, as it contributes to the debate over evolutionary forces maintaining CpG islands in mammalian genomes, including the human genome. The paper is clearly interesting. I have three comments:

1) The authors state that the rate of C > T transitions within CpG contexts is elevated 3–12 fold.

I believe that the effect is much stronger and of the order of 15 fold.

Response: *We made the following clarification to the text:*

“Frequent cytosine methylation is usually considered as the main cause of a 3-12-fold excess of C > T substitutions in the CpG context [4–7] (estimates of *de novo* mutation rates have shown an up to 18-fold excess [8]), resulting in a significant underrepresentation of CpG dinucleotides in mammalian genomes, including the human genome [5, 6]”.

2) The methylation data come from mature sperm cells. The most relevant cell type is spermatocytes. More generally, methylation of all cell types in spermatogonia and oocyte lineages are relevant. I understand that there is nothing the authors can do about it. However, acknowledging this level of complexity warrants a separate longer discussion.

Response: *We completely agree with the reviewer on this point and we tried to discuss this in more detail in the introduction since selection of the cell types in crucial for the paper and is worth discussion from the beginning.*

“What methylation data is appropriate for this study? Only germline mutations (mutations in gametes, zygotes, blastomeres, embryoblast cells, epiblast cells, primordial germ cells, and gametogonia) can be inherited, while somatic cell mutations cannot. Therefore, only the methylation patterns in former cell types are relevant to our study. Germline cells undergo many division cycles on the development path from zygotes to gametogonia, and therefore are likely to accumulate many mutations, including methylation-dependent CpG > TpG mutations. While somatic methylation itself should not affect observed substitution rates, although it can be correlated with mutation rates in regions where somatic and germline methylation profiles are similar [16, 17].

Consistent with previous views [18], a recent study confirmed that the number of mutations in human offspring is highly correlated with their fathers’ age [8], suggesting prevalent accumulation of mutations in the male germline. Also sperm cells demonstrate one of the highest methylation levels among the germline cells [17]. This makes sperm cells one of the most promising objects to study methylation-dependant substitution rates [17]. Fortunately, high quality methylation data are available for human sperm [9, 19, 20]. Spermatogonia cells would be an even better choice for the purpose of our study, since they constantly divide by mitosis during the entire male adult life accumulating a perceptible fraction of all germline mutations. Unfortunately, genome-wide methylation profiles of spermatogonia are currently unavailable. Oogonia methylation might also contribute to mutation rates, but such data is also unavailable. Oocytes have been shown to exhibit higher CGI methylation levels than sperm [20], but they do not undergo cell division and thus are not expected to acquire many mutations. Also, there is available data on methylation in embryonic stem cells (ESC), another type of germline cells undergoing many mitotic divisions [10]. Thus sperm and ESC data seem to be the most relevant available approximations of methylation patterns in the human germline.”

3) It would be great to extend this work to the analysis of allele frequency distribution. I understand that such analysis may be underpowered with currently available datasets. In a similar vein, growing datasets of de novo mutations offer a possibility to analyze de novo mutations directly as opposed to human chimpanzee divergence. Again, the author may find currently available datasets underpowered for such analysis.

Response: *We sincerely appreciate reviewer’s suggestion. In fact, in previous studies we tried to perform allele frequency test but failed due to the lack of data. Yet, in the last year, a lot of new data were released and we managed to obtain enough data for statistically significant conclusions. We compared the allele distributions of CpGs within and outside CGI and demonstrated the decreased frequencies of derived alleles in GGIs. We added the following paragraph to the results and appropriate changes to methods.*

***“The frequencies on derived alleles for C/T and G/A polymorphisms are decreased for methylated CpGs in CGIs as compared to methylated non-CGI controls.***

Using allele frequency data obtained within the 1000 genome project (see Methods: Allele frequency analysis) we evaluated the hypothesis that there is excessive negative selection on methylated CpGs within CGIs as compared to non-CGI genomic regions. The prediction of this hypothesis is that smaller allele frequency values should be observed for derived T and A alleles within CGIs. We observed a highly significant although small effect in support of this hypothesis. Out of two sets of 241526 methylated CpG dinucleotides 16844 and 41360 contained polymorphisms for the CGI and non-CGI datasets respectively. The average derived allele frequency were 0025 and 0029 respectively and the median values were 0,0018 and 0,0023 respectively (*P* = 2.2e-16, Wilcoxon rank sum test). Similar median values (0.0018 and 0.0023 respectively) were obtained for derived allele frequencies for CGI and non-CGI intergenic regions, thus the effect was not due to selection on the gene level.”

We also made changes in abstract and conclusions.

“Our findings are consistent with strong negative selection preserving methylated CpGs within intergenic CGIs, yet reduced CpG > TpG mutations rates in CGIs cannot be fully eliminated.”

“Although it can be hypothesized that the ^5m^CpG > TpG substitution rate itself is lower within CpG islands due to reduced mutation rates, our data suggests strong negative selection acting within CGIs. In any case we can conclude that the likelihood of nucleotide substitutions, including those associated with diseases, depends on the presence of CGIs whether methylated or not”.

We also explained in more details why human *de novo* mutations analysis cannot be performed with the current data.

“Using published data on human *de novo* mutations would be a more direct approach [8], however, the observed number of *de novo* mutations in CGIs is currently too small, making the statistical analysis under-powered. This occurs because CGIs cover less than 1 % of the genome [15]. On average one out of 20 dinucleotides (roughly estimated) within CGIs are CpGs and not not all of them are methylated.”

### Reviewer 4 (Alexey Kondrashov)

Review of “Preservation of methylated CpG dinucleotides in human CpG islands” by A. Y. Panchin et al. I like the paper. Indeed, a priori there are two mainstream explanations for the high local prevalence of an apparently mutagenic context reduced mutation rate or negative selection. The mutational explanation for some reason is widely accepted for CpG islands, although it can no longer be viewed as null hypothesis, because we know that a lot of noncoding sequences are under selection in mammals. Thus, I am surprised that nobody seriously tested the alternative, selective explanation before but, as far as I know, the results reported are novel. Using patterns of methylation is spermatozoids definitely makes sense. The paper is well written, and I have only small comments. p. 3. Paternal age effect was not discovered by Kong et al. It was well known decades earlier (see Crow PNAS 94, 8380, 1997). p. 3.

Response: *We appreciate the reviewer’s evaluation of our work. Indeed, starting this work we were extremely surprised that nobody has done it before.*

We corrected the statement about paternal age.

“Consistent with previous views [18], a recent study confirmed that the number of mutations in human offspring is highly correlated with their fathers’ age [8], suggesting prevalent accumulation of mutations in the male germline.”

These days it is a common practice to provide an additional Abstract at the bottom of an Introduction. Still, it makes no sense.

Response: *We removed the summary from the Introduction to avoid repetition of the results and conclusions.*

p. 4 (bottom) Conversion occurs between double stranded DNAs.

Response: *We corrected the statement*

“In mammals gene conversion occurs between double-stranded DNA and is biased towards increasing GC content [25], most likely placing C or G when a mismatched pair is observed”.

p. 7 (top). The authors should be commended for generally calling substitutions substitutions. Here, however, they call them mutations which, of course, undermines their key thesis that substitution is not equivalent to mutation. A. Kondrashov.

Response: *We double-checked for the presence of such misused terms keeping the word mutation only in places of the text where we explicitly mean it.*

## Additional files

Additional file 1: Figure S1. Flowchart of the case/control CpG dinucleotide selection, using sperm methylation data. For the two other cell types, the process was similar, but only the methylated/unmethylated comparison was performed for reasons stated in Result and Discussion: “Cytosine methylation in sperm cells affects CpG > TpG substitution rates both within and outside of CpG islands”. (PDF 9 kb) Additional file 2: Table S1.
^5m^CpG > TpG substitution rates in various regions of the genome. The CGI impact column represents the excess of ^5m^CpG > TpG substitutions outside CGIs as compared to within CGI regions. To avoid potential bias, we did not split the sample and control CpGs into gene-associated and intergenic regions (or hotspots/no hotspots regions). We first split all available CpGs into gene-associated or intergenic regions and then we searched for appropriate controls within the same regions. If we could not find a control, we excluded the sample CpG from consideration, resulting in the reduced number of sample CpGs. (DOC 59 kb)

## Abbreviations

BGC: biased gene conversion
CGI: CpG island
MBD: methyl-binding domain proteins
SNP: single nucleotide polymorphisms
TFBS: transcription factor binding site
