# Supplementary figures and images for: Preservation of methylated CpG dinucleotides in human CpG islands
Source: Biol Direct. 2016 Mar 22;11:11. doi: 10.1186/s13062-016-0113-x (PMC4804638; doi:10.1186/s13062-016-0113-x)

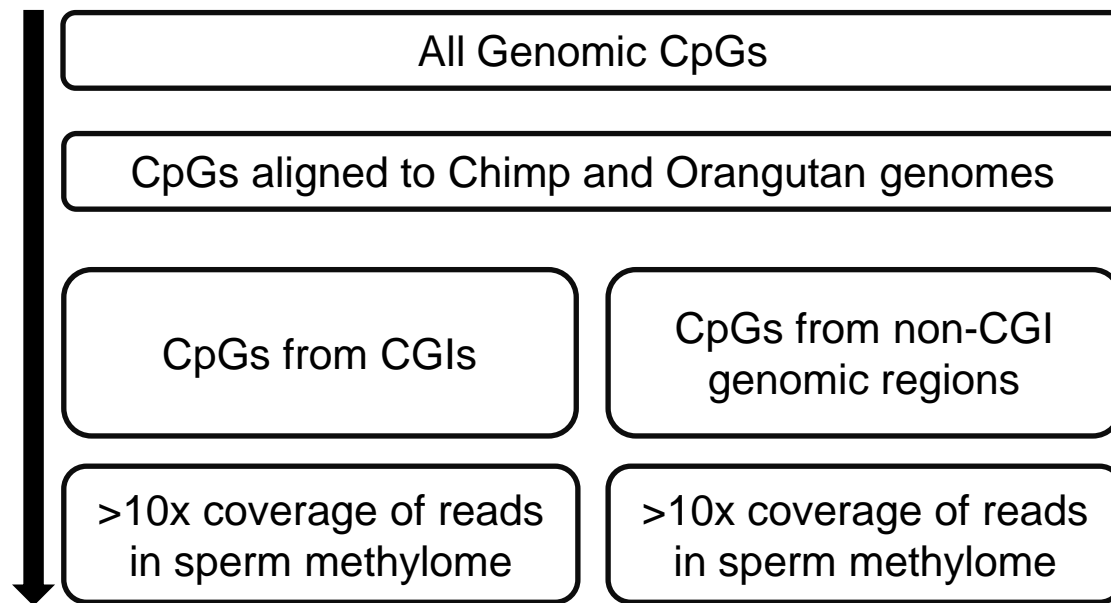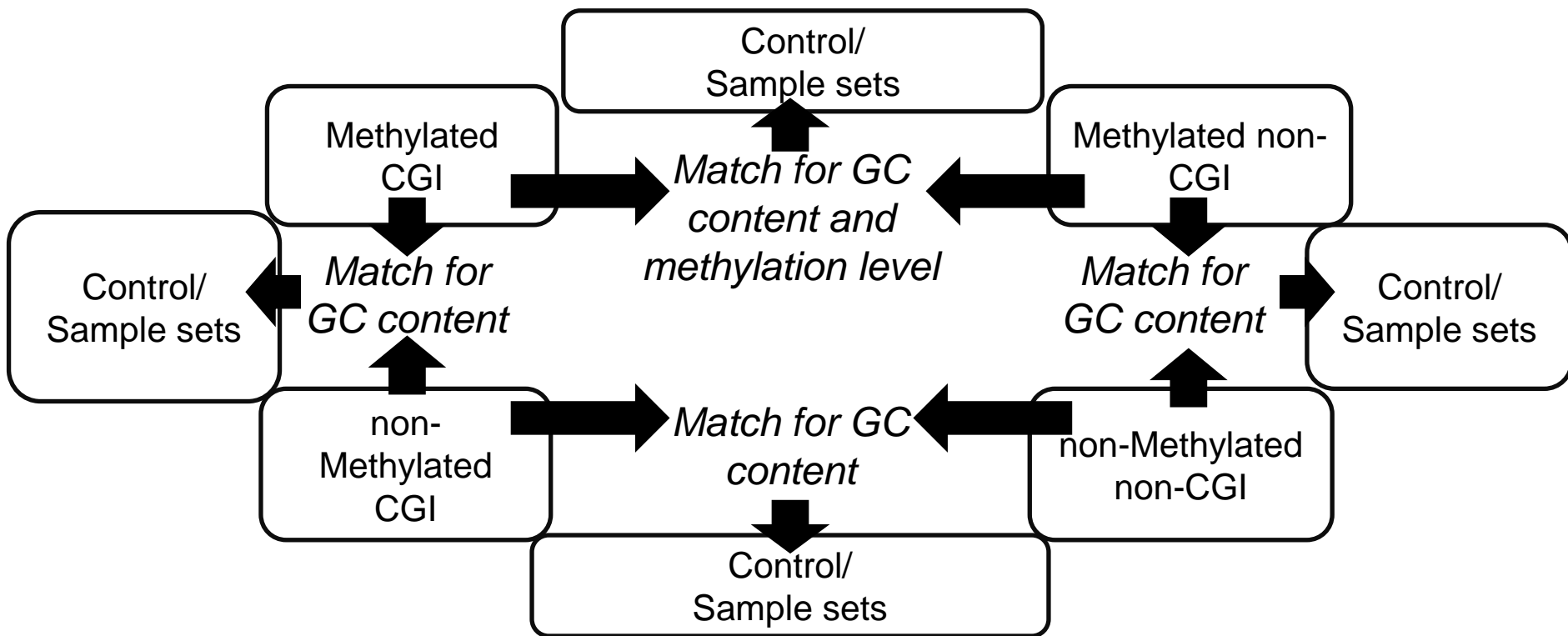

Supplement: Additional file 1: Figure S1. — Flowchart of the case/control CpG dinucleotide selection, using sperm methylation data. For the two other cell types, the process was similar, but only the methylated/unmethylated comparison was performed for reasons stated in Result and Discussion: “Cytosine methylation in sperm cells affects CpG > TpG substitution rates both within and outside of CpG islands”. (PDF 9 kb) [file 13062_2016_113_MOESM1_ESM.pdf]
