# Supplementary material for: Preservation of methylated CpG dinucleotides in human CpG islands
Source: Biol Direct. 2016 Mar 22;11:11. doi: 10.1186/s13062-016-0113-x (PMC4804638; doi:10.1186/s13062-016-0113-x)
Supplement: Additional file 2: Table S1. — 5mCpG > TpG substitution rates in various regions of the genome. The CGI impact column represents the excess of 5mCpG > TpG substitutions outside CGIs as compared to within CGI regions. To avoid potential bias, we did not split the sample and control CpGs into gene-associated and intergenic regions (or hotspots/no hotspots regions). We first split all available CpGs into gene-associated or intergenic regions and then we searched for appropriate controls within the same regions. If we could not find a control, we excluded the sample CpG from consideration, resulting in the reduced number of sample CpGs. (DOC 59 kb) [file 13062_2016_113_MOESM2_ESM.doc]

Additional file 2: Table S1.

| Genomic regions | Methylation status | CpG>TpG substitution rate within CGI: rate (absolute values mutated/total) | CpG>TpG substitution rate outside CGI : rate (absolute values mutated/total) | | CGI impact |
| --- | --- | --- | --- | --- | --- |
| **Case/control analysis of the methylation impact** | | | | | |
| **ESC** | | | | | |
| Whole genome | Methylated | 0.080  (4288/53424) | 0.166  (24419/147425 | Not applicable | |
|  | Unmethylated | 0.025  (1362/53424) | 0.159  (23406/147425) | Not applicable | |
| **Fibroblasts** | | | | | |
| Whole genome | Methylated | 0.089  (11179/125165) | 0.167  (79442/475948) | Not applicable | |
|  | Unmethylated | 0.038  (4806/125165) | 0.149  (70816/475948) | Not applicable | |
| **Sperm** | | | | | |
| Whole genome | Methylated | 0.086  (30844/357140) | 0.246  (29215/118686) | | Not applicable |
|  | Unmethylated | 0.027  (9465/357140) | 0.053  (6610/118686) | | Not applicable |
| **Case/control analysis of the CGI impact** | | | | | |
| **Sperm** | | | | | |
| Whole genome | Methylated | 0.110  (26654/241526) | 0.227  (54752/241526) | | 2.064 |
|  | Unmethylated | 0.031  (3627/117033) | 0.046  (5388/117033) | | 1.484 |
| Hotspots | Methylated | 0.135  (1196/8837) | 0.257  (2268/8837) | | 1.904 |
|  | Unmethylated | 0.035  (163/4671) | 0.042  (198/4671) | | 1.200 |
| No hotspots | Methylated | 0.109  (25328/232331) | 0.225  (52355/232331) | | 2.064 |
|  | Unmethylated | 0.031  (3899/124068) | 0.050  (5704/124068) | | 1.484 |
| RefSeq genes | Methylated | 0.117  (15174/130000) | 0.227  (29553/130000) | | 1.940 |
|  | Unmethylated | 0.032  (2119/65993) | 0.045  (2952/65993) | | 1.406 |
| No Refseq genes | Methylated | 0.103  (11382/110863) | 0.223  (24740/110863) | | 2.165 |
|  | Unmethylated | 0.031  (1953/62737) | 0.046  (2879/62737) | | 1.484 |
| **Sperm, only non-genic regions** | | | | | |
| Conserved TFBS | Methylated | 0.040  (991/24517) | 0.130  (3370/24517) | | 3.224 |
| MBD regions, donor 1 | Methylated | 0.185  (655/3547) | 0.175  (615/3547) | | 0.950 |
| MBD regions, donor 2 | Methylated | 0.181  (759/4187) | 0.171  (1001/4187) | | 0.942 |
| H3K4me1 | Methylated | 0.033  (9/273) | 0.116  (25/273) | | 3.527 |
| H3K4me3 | Methylated | 0.040  (275/6891) | 0.075  (258/6891) | | 2.610 |
| H3K27me3 | Methylated | 0.037  (214/5730) | 0.097  (201/5730) | | 1.888 |
| Histone Mnase | Methylated | 0.036  (205/5635) | 0.124  (635/5635) | | 3.402 |
